# Efficacy and Safety of Biologics and Small Molecules for Moderate-to-Severe Hidradenitis Suppurativa: A Systematic Review and Network Meta-Analysis

**DOI:** 10.3390/pharmaceutics15051351

**Published:** 2023-04-28

**Authors:** Ya-Chu Tsai, Chen-Yiu Hung, Tsen-Fang Tsai

**Affiliations:** 1 Department of Dermatology, Far Eastern Memorial Hospital, New Taipei 220, Taiwan; gracybamboo@gmail.com; 2 Department of Fashion Styling and Design, Minghsin University of Science and Technology, Hsinchu 30401, Taiwan; 3 Department of Thoracic Medicine, Chang Gung University College of Medicine, Chang Gung Memorial Hospital at Linkou, Taoyuan 333, Taiwan; 4 Department of Dermatology, National Taiwan University Hospital and National Taiwan University College of Medicine, Taipei 100, Taiwan

**Keywords:** hidradenitis suppurativa, biologic agents, small molecular drugs, HiSCR, DLQI, adverse effects

## Abstract

**Background:** Treatment of hidradenitis suppurativa (HS) is difficult and current guidelines are based mainly on expert opinion and non-randomized controlled trials. Recently, there have been some targeted therapies using uniform primary endpoints for outcome assessment. **Objective:** Recommendations can be provided on selecting biologics and targeted synthetic small molecules for refractory HS by comparing the efficacy and safety of these medications. **Methods:** Databases including ClinicalTrial.gov, Cochrane Library, and PubMed were searched. Randomized controlled trials (RCTs) for moderate-to-severe HS were eligible. We performed random-effect network meta-analysis and ranking probability. The primary outcome was Hidradenitis Suppurativa Clinical Response (HiSCR) at 12–16 weeks. Secondary outcome included Dermatology Life Quality Index (DLQI) 0/1, mean change of DLQI from baseline, and adverse effects. **Results:** A total of 12 RCTs involving 2915 patients were identified. Adalimumab, bimekizumab, secukinumab 300 mg q4w and secukinumab 300 mg q2w showed superiority to placebo in HiSCR at weeks 12 to 16. In addition, there was no significant difference between bimekizumab and adalimumab as measured by HiSCR (RR = 1.00; 95% CI: 0.66–1.52) and DLQI 0/1 (RR = 2.40, 95% CI: 0.88–6.50). In terms of ranking probability for achieving HiSCR at 12–16 weeks, adalimumab ranked first, followed by bimekizumab, secukinumab 300 mg q4w, and secukinumab 300 mg q2w. All biologics and small molecules did not differ in the development of adverse effects compared to placebo. **Conclusions:** Adalimumab, bimekizumab, secukinumab 300 mg q4w and secukinumab 300 mg q2w represent four regimens that produce better outcomes than placebo without increased risk of adverse events. Adalimumab and bimekizumab exhibited best HiSCR and DLQI 0/1 between weeks 12–16.

## 1. Introduction

Hidradenitis suppurativa (HS), also called acne inversa, is a chronic, inflammatory, recalcitrant skin follicular disease [1]. The overall prevalence is around 0.4% and onset usually occurs after puberty [2]. Lesions are characterized by deep-seated nodules, abscesses, forming draining sinus tracts, and leaving bridge scars. The course typically remains quiescent for a period of time, followed by an unexpected acute exacerbation, and the cycle is repeated again. HS mostly located on flexural sites such as axilla, inframammary, inguinal, and anogenital areas. Symptoms including pruritus, pain, and bad odor cause severe discomfort, low self-esteem, and poor quality of life.

HS may also trigger systemic inflammation and affect the function of other organs. Studies have suggested the presence of associated comorbidities including metabolic syndrome (hypertension, hyperlipidemia, diabetes mellitus, and obesity), inflammatory bowel disease, polycystic ovarian syndrome, spondyloarthritis, psychiatric disorder, and psoriasis [3,4,5,6,7].

Diagnosis of HS is primarily based on lesion characteristics and clinical course. However, ultrasound helps to detect subclinical features and provide more accurate treatment plans. In a recent study, sinus tracts were subclassified into type A to D via sonography, depending on whether there was destruction of the dermo-epidermal junction and treatment response was found to correlate with these subtypes [8]. Ultrasound has also been utilized to assess the effectiveness of intralesional triamcinolone for treating fistulas and to monitor the response of clindamycin with rifampicin for disease regression [9]. Additionally, Color Doppler ultrasound can provide information regarding the vascular distribution (peripheral, central or mixed) and vascular degree (minimal, moderate, or high), which was applied to observe the response of adalimumab in an HS study [10].

Genetic variants of HS are complex and heterogenous among the three phenotypes: familial, sporadic and syndromic. Genetic mutations include gamma-secretase, autoinflammatory and keratinization genes. Approximately 30–40% of patients have a positive family history [11]. However, gamma-secretase gene mutations only account for a small proportion of cases. In addition, mutations of gamma-secretase genes alone are insufficient to trigger HS. This implies that susceptibility genes precipitating the onset of HS have not yet been fully identified [12]. The integration of genomics, transcriptomics, and epigenomics through OMICS approaches might present a better way to comprehensively understand the disease.

The pathogenesis of HS is multifactorial, involving various factors such as genes, environment, microbiota, and hormones to varying degrees. After exogenous stimulation, innate immune reactions are activated around the follicles in patients with genetic predisposition. This causes follicular hyperkeratosis leading to infundibulum occlusion and follicular dilatation. The dilated follicles rupture and next recruit Th1 cells, Th17 cells, B cells, neutrophils, and macrophages to the skin. These immune cells release several pro-inflammatory cytokines: TNF-α, IL-17, IL-1β, IL-12, IL-23, IL-36, and CXCL/IL-8 and contribute to the formation of inflammatory nodules and abscesses. In a chronic state, fibrotic factors become involved to create sinus tracts and scarring [4,13].

With the understanding of immunopathogenesis, targeted therapies have gained substantial attention on HS. Among all cytokines, TNF-α and IL-17 are considered to play major roles. Adalimumab, a TNF-α inhibitor, represents the first and only Food and Drug Administration (FDA)- and European Medicines Agency (EMA)-approved medication for moderate-to-severe HS to date. However, there is still a number of people who are unable to achieve treatment goals and experience high recurrence rates. The term “therapeutic delay” refers to the time elapsed from the first onset of HS to the initiation of adalimumab treatment. A therapeutic delay of 10 years showed significantly poor response to adalimumab, especially at week 16. The evidence of “window of opportunity” underscores the importance of early initiation of adalimumab to achieve better clinical outcomes [14]. Some anti-IL-17 inhibitors have recently finished phase III trials. Other potential classes of drugs with high-quality randomized controlled trials (RCTs) were also included in our analysis. This article aimed to provide recommendations on selecting targeted therapies for HS by simultaneously comparing more than two interventions via network meta-analysis (NMA) [13,15,16].

## 2. Methods

### 2.1. Protocol

This NMA was performed and reported in accordance with the guidelines of Preferred Reporting Items for Systematic Reviews and Meta-analyses Extension Statement for Network Meta-Analyses (PRISMA-NMA) [17]. The systematic review was registered on the PROSPERO database with the number CRD42023400921.

### 2.2. Search Strategy

We searched electronic database including PubMed, ClinicalTrial.gov, and Cochrane Library for relevant studies of RCTs from their receptive inception to 19 January 2013. References from the eligible studies were hand-searched for additional pertinent articles.

### 2.3. Eligibility Criteria and Study Selection

Inclusion criteria were as follows: (1) Placebo- or active comparator-controlled human studies. (2) Trials focusing on the efficacy and safety of biologic agents or small molecule drugs. (3) RCTs with at least one of the following outcomes reported: Hidradenitis Suppurativa Clinical Response (HiSCR), Dermatology Life Quality Index (DLQI), and adverse events. However, single-arm studies, retrospective studies, cross-sectional studies, review articles, observational studies and case series were excluded. Reports regarding an open-label phase or a post hoc analysis were not included either.

Two of the authors (Y.C. Tsai and C.Y. Hung) independently screened all titles plus abstracts after deduplication, and scrutinized full texts to identify potential trials. Any disagreement was resolved by discussion with a third author (T.F. Tsai).

### 2.4. Risk of Bias Evaluation and Data Extraction

The extracted data of interest contained baseline demography (age, gender, body mass index, and severity), the number of participants, treatment regimens, and outcome evaluation. Our primary outcome was HiSCR. Secondary outcomes were DLQI 0/1, mean DLQI change from baseline, and adverse effects.

The quality of enrolled studies was appraised by two investigators (Y.C Tsai and C.Y. Hung) using the revised Cochrane risk-of-bias tool for RCTs [18]. Through evaluating methodology, the overall risk of bias was judged as low, high, or some concern. If discrepant opinion existed, the third author (T.F. Tsai) was consulted for arbitration.

### 2.5. Data Synthesis and Statistical Analysis

For clinical applicability, only trials with sample size greater than 20 subjects were included in the NMA. This study contained pharmacological interventions of adalimumab, secukinumab, bimekizumab, avacopan, risankizumab, guselkumab, IFX-1, and CJM 112. The NMA was conducted via ShinyNMA Version 1.01 (https://jerryljw.shinyapps.io/ShinyNMA_/ (accessed on 14 February 2023)) [19]. It is an online free-to-use cloud computing NMA for researchers and is in accordance with the requirements of PRISMA 2020 for reporting [20]. The analysis tool was R software (version 4.1.0) and R packages: metafor (version 2.4-0), netmeta (version 1.3-0), BUGSnet (version 1.0-4). Binary outcomes (HiSCR, DLQI 0/1, and adverse effects) and a random-effect model were performed, and the results were presented as risk ratio (RR) with 95% confidence interval (CI). Continuous outcomes (mean DLQI change from baseline) were presented as mean difference (MD) with 95% CI. Moreover, ranking probability was analyzed for HiSCR and DLQI 0/1 and reported as P score. The P score represents the extent of certainty that one therapy is superior to other therapies. The higher the score means the better efficacy than others. Furthermore, inconsistency assessment was performed by using loop-specific heterogeneity and we also compared the effect sizes between pairwise meta-analysis and NMA.

## 3. Results

### 3.1. Search Result

**Figure 1:**
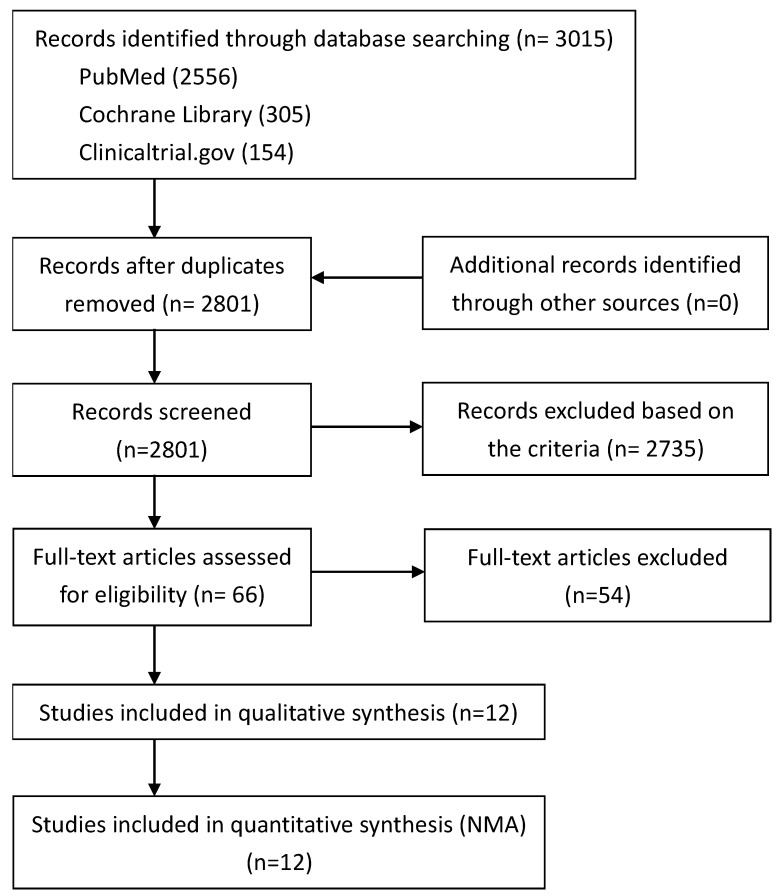
PRISMA flow diagram of article selection.

A total of 3015 articles were identified in the initial search of databases. After we removed the duplicates and screened the titles and abstracts, 66 articles were proceeded to the full-text evaluation. Lastly, 12 RCTs were eligible for qualitative and quantitate analysis. The details of search steps were shown in Figure 1, and the characteristics of the included RCTs are listed in Table 1.

Among the RCTs, 7 classes of biologics (adalimumab, secukinumab, bimekizumab, risankizumab, guselkumab, IFX-1, and CJM112) and one small molecule inhibitor (avacopan) with a total sample size of 2915 patients were included in the NMA [21,22,23,24,25,26,27,28,29,30,31]. The mean age ranged from 32.0 to 40.7 years old, women comprised a greater proportion of the sample (50 to 81%), body mass index showed obese status (31.3 to 37.3), and baseline Hurley stage II accounted for 48.0 to 67.2%.

Apremilast, anakinra, MABp1, and INCB054707 were not enrolled in the statistical analysis due to small sample sizes. In addition, infliximab and etanercept were excluded in our research because there were no suitable outcomes to analyze according to the criteria.

**Figure 2:**
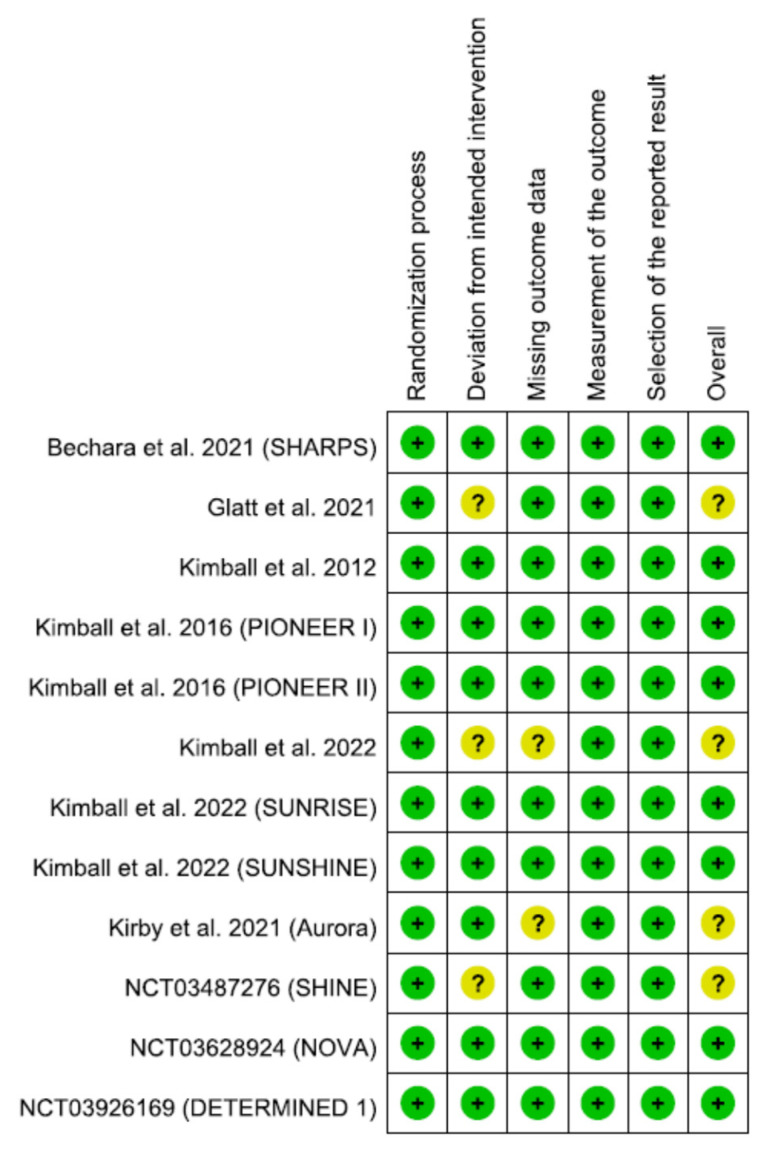

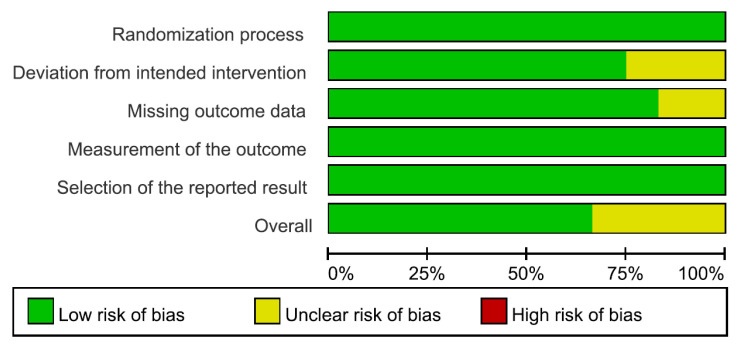
Summary of risk-of-bias assessment [21,22,23,24,25,26,27,28,29,30,31].

Most studies showed overall low risk following thorough evaluation of risk of biases. Of the studies, four had some concern, and none were judged as high risk. Moreover, three studies adopted per-protocol or full-set analysis rather than intention-to-treat in managing the deviation from the intended intervention, and thus we judged them as some concern. On the other hand, two studies were appraised as some concern in processing the missing data (Figure 2) (Supplementary Table S1).

### 3.2. Primary Outcome: HiSCR at 12–16 Weeks

**Figure 3:**
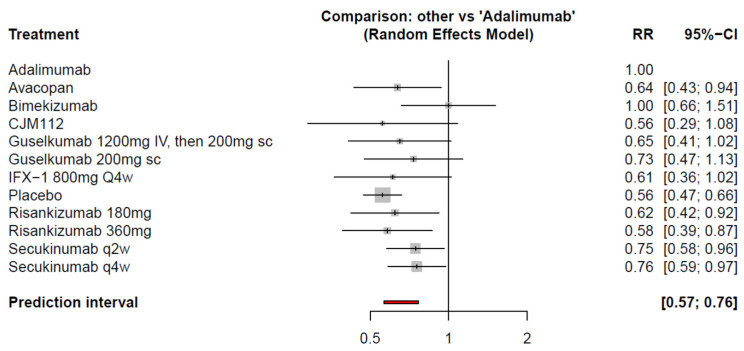
Comparison of HiSCR between adalimumab and active comparators at weeks 12–16.

Table 2 demonstrates the outcome of pairwise meta-analysis and NMA of HiSCR at weeks 12 to 16. The results presented in the league table reveal that significantly higher odds of HiSCR50 were achieved by adalimumab, bimekizumab, secukinumab every four weeks (q4w) and secukinumab q2w compared with placebo. In comparison with adalimumab, it showed significantly better efficacy than secukinumab q4w and q2w, avacopan, and risankizumab 180 mg and 360 mg, as displayed in Table 2 and Figure 3. With regard to CJM 112, guselkumab, and IFX-1, adalimumab had numerically higher chance of achieving HiSCR, but statistical significance was not achieved. In addition, bimekizumab was the only enrolled medication numerically and statistically not inferior to adalimumab assessed by HiSCR (RR = 1.00; 95% CI: 0.66–1.51).

**Figure 4:**
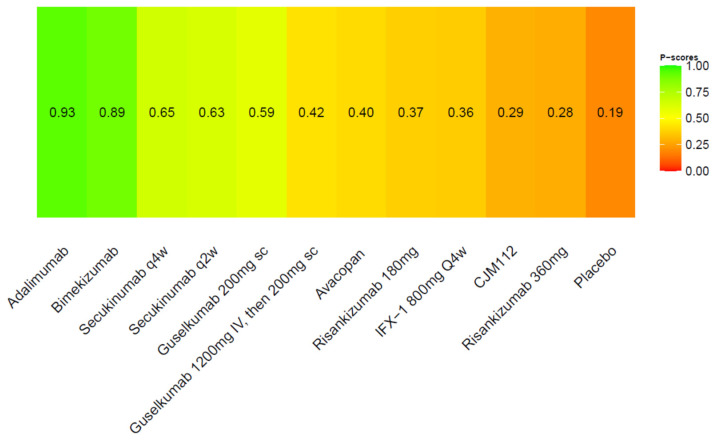
Ranking probability of HiSCR.

We further conducted a ranking probability analysis. The ranking of HiSCR indicated that adalimumab was the most effective, followed by bimekizumab, secukinumab q4w, and secukinumab q2w (Figure 4).

### 3.3. Secondary Outcomes: DLQI 0/1, Mean DLQI Change, and Adverse Effects at 12–16 Weeks

Of the eight study medications, two reported on DLQI 0/1 response and three offered sufficient data for mean DLQI change analysis.

#### 3.3.1. DLQI 0/1

**Figure 5:**
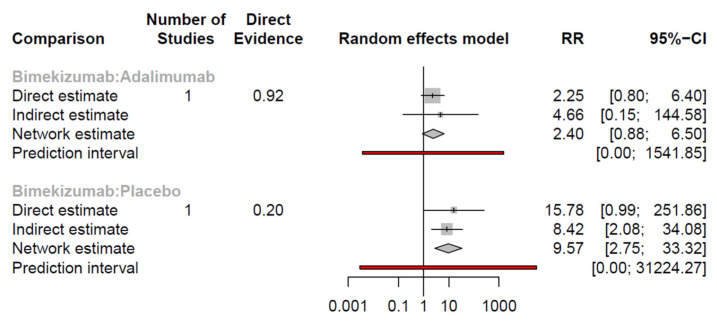
Integrated evidence of DLQI 0/1. (1) Bimekizumab versus adalimumab. (2) Bimekizumab versus placebo.

**Figure 6:**
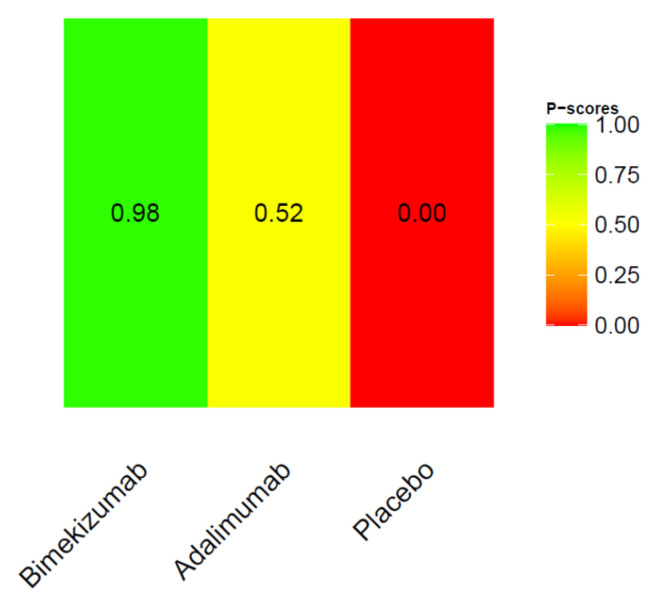
Ranking probability of DLQI 0/1.

Both bimekizumab and adalimumab had higher probability of exhibiting DLQI 0/1 at week 12 to 16 compared with placebo according to the NMA. However, there was no significant difference between bimekizumab and adalimumab (Table 3, Figure 5). The ranking probability in Figure 6 showed bimekizumab was the most effective in achieving DLQI 0/1, followed by adalimumab and placebo.

#### 3.3.2. Mean Change of DLQI from Baseline

Guselkumab 200 mg sc was superior to placebo in amelioration of life quality as measured by mean change of DLQI from baseline to weeks 12–16, while CJM112, guselkumab 1200 IV loading, and IFX-1 were not (Table 4). There were insufficient data for conducting NMA of DLQI among secukinumab, risankizumab, and avacopan.

#### 3.3.3. Adverse Events

Safety outcomes were measured by any adverse events or treatment-emergent adverse effects, which were defined in the trials as onset or exacerbation of an adverse event from the first dose of a study medication until a period of time after the last dose (ranging from 70 to 140 days). The NMA league table of adverse events is shown in Table 5. All the biologics and small molecules revealed no significant difference to the placebo and among all treatment comparisons in developing adverse events.

### 3.4. Network Consistency

**Figure 7:**
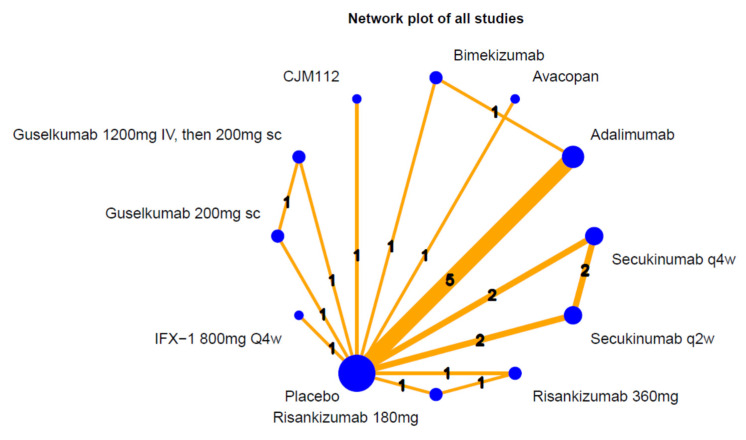
Network plot of biologics and small molecular drugs for HS.

With respect to loop-specific heterogeneity, there was no inconsistency between indirect and direct comparisons for all efficacy and safety outcomes. Table 6 illustrates the results of inconsistency evaluation of HiSCR. In addition, forest plots in Figure 7 reveal that the differences between pairwise meta-analysis and NMA were all insignificant.

## 4. Discussion

In the present NMA, adalimumab, bimekizumab, and secukinumab q2w and q4w achieved significantly better response than placebo as per HiSCR at 12 to 16 weeks. Regarding adverse events, none of the therapies bore higher risks than placebo. There was no significant difference between bimekizumab and adalimumab in HiSCR and DLQI 0/1. However, the ranking probability suggested bimekizumab as the most effective in ameliorating the quality of life, followed by adalimumab; while adalimumab ranked first concerning the HiSCR, followed by bimekizumab, secukinumab q4w, and secukinumab q2w.

Although the key cytokine leading the outbreak and recalcitrant course of HS is still not definitively known, significantly elevated TNF-α and IL-17 levels in HS lesions compared with healthy control and psoriasis were noticed [8,32]. IL-1β, IL-6, IL-8, IL-12, IL-23, IL-36, and IFN-γ were also found to be related to HS development [16,32,33]. Our systematic review included TNF-α inhibitors (adalimumab, infliximab, etanercept), IL-1 inhibitors (anakinra, MABp1), IL-17 inhibitors (bimekizumab, secukinumab, CJM 112), IL-23 inhibitors (guselkumab, risankizumab), a phosphodiesterase-4 inhibitor (apremilast), a Janus kinase 1 inhibitor (INCB054707), and complement C5a or C5a-receptor inhibitors (avacopan, IFX-1) (Supplementary Table S2) [34,35,36,37,38,39]. Among them, eight of the biologics and small molecule agents were eligible for quantitative analysis.

At least 30 evaluation instruments for the treatment efficacy of HS had been proposed. HS Severity Index (HSSI), modified Sartorius score (mSS), physician global assessment (PGA), visual analogue scale (VAS), DLQI, HS-PGA, and the refined Hurley staging system were some of the instances used in RCTs. Nevertheless, 90% of them were not well-validated and most of these tools were not originally designed for measuring the HS treatment response [8,40]. HiSCR was the minority with good-quality validation for assessing the inflammation control of the interventions and its adoption is becoming a trend as a primary endpoint for clinical trials [41]. Except for objective physician-reported efficacy, subjective patient-reported outcomes (such as DLQI or VAS score) should be integrated into the overall measures suggested by HS ALLIANCE in 2018 because patients could provide insights on symptoms, daily activity, psychological level, and sociality [6]. These were the reasons we selected HiSCR and DLQI as primary and secondary outcomes for NMA. In the guselkumab 200 mg sc group, significant improvement was achieved in mean DLQI change but not in HiSCR compared with the placebo group [28]. The discordance may arise from the inherent difference between a subjective and an objective measurement or may be due to the non-specific instrument, DLQI, for HS evaluation.

An NMA published in 2022 enrolled not only four biologics but also botulinum toxin, oral tetracycline, and clindamycin for HS treatment [42]. However, baseline disease severity was heterogenous and ranged from mild to severe, and the outcome of HiSCR was not included. They concluded that botulinum toxin B was statistically more effective than adalimumab with regard to quality of life, but it was aimed for mild HS. In addition, the regimen of adalimumab in this comparison was low dose rather than the approved higher loading dose, weekly regimen for HS. In our NMA, HiSCR was set as the major outcome since most RCTs of biologics and small molecule agents had reported this common response result. Infliximab was measured by HSSI and thus was excluded [34]. Meanwhile, apremilast, anakinra, MABp1, INCB054707, and etanercept were not incorporated in the NMA because trials with a sample size less than or equal to 20 patients would have a higher probability of statistical error and less clinical practicality. In fact, several examples of targeted therapies with ≤ 20 participants showing good response in phase II trials had failed in phase III large-scaled trials in inflammatory diseases [43,44]. In addition, we focused on moderate-to-severe HS since this represents the inclusion criteria of most trials in which non-biologic therapies, such as topical or systemic antibiotics, were not allowed.

Another meta-analysis (Huang at al.) depicted bimekizumab and adalimumab as the only two biologics that performed better than placebo as measured by HiSCR, which was in line with our finding [45]. However, we added on secukinumab q2w and q4w, of which the phase 3 trial results were released recently, as another choice for HS. Additionally, we presented another result of NMA by using adalimumab, currently the only licensed medication for HS, as a comparator. It showed adalimumab was superior to secukinumab as well as bimekizumab, but there was no statistical difference between adalimumab and bimekizumab in terms of HiSCR at weeks 12 to 16. With regard to DLQI 0/1, Huang et al. revealed bimekizumab did not significantly outweigh placebo. By contrast, NMA in our study demonstrated bimekizumab outperformed the placebo (RR = 9.57, 95% CI: 2.75–33.32). Although our pairwise meta-analysis indicated similar results to Huang’s, the confidence interval was very close to 1 (RR = 15.78, 95%CI: 0.99–251.86). The previous published study only conducted a meta-analysis, which lacked indirect comparison. The direct evidence could be compensated by indirect evidence and thus often offered more reliable data. NMA could provide an advantage of more precise estimation of interventions than an open loop of a single direct comparison or a single indirect comparison. There was no significant inconsistency between the direct and indirect comparisons in our study concerning bimekizumab versus placebo in DLQI 0/1 (Table 6). The differences between the pairwise meta-analysis and NMA were also not significant (Figure 7).

Emerging targeted therapies for HS focuses much attention on the pathogenesis and most of them are currently under the investigation of phase II clinical trials. Anti-CD40 (iscalimab), anti-Leukotriene A4 (LYS006), CXCR1/2 (LY3041658), anti-IL36 (imsidolimab, spesolimab) and kinase inhibitors (upadacitinib, PF-06650833, brepocitinib, ropsacitinib) are the ones other than the above-mentioned targets which have no outcome of RCTs available so far [15,16].

## 5. Limitations

There are some limitations of our article. First, some of our enrolled medications had only one RCT with uploaded data eligible for conducting the NMA. With the appearance of rising numbers of phase II and phase III trials regarding the biologic agents and small molecular drugs for refractory HS, more participants and numbers of RCTs would be able to offer more information for making dynamic adjustments when choosing a targeted therapy. However, to the best of our knowledge, this is the first NMA that focuses on biologics and small molecules for HS. We provided a statistical index for which targeted therapy to initiate and how to evaluate the outcome. Second, the existing evidence covered a period spanning from 12 to 16 weeks. Therefore, long-term efficacy and safety data still need further evaluation. It had previously been reported that HiSCR could be reached after 24 weeks if initial quick response was not achieved or partially achieved as for the patients with dermal tunnels [46]. Further subgroup analysis is needed to determine the appropriate timing for switching or stopping biologic agent treatment due to the varying peak response times of different HS phenotypes. Third, the combination therapy of targeted drugs plus surgery, topical agents, or systemic anti-inflammatory agents is out of the scope of our study. Here, we highlight research gaps in the literature and unmet needs for investigation.

## 6. Conclusions

In conclusion, our analysis suggested that among all biologics and small molecular agents, adalimumab, bimekizumab, and secukinumab were effective for moderate-to-severe HS. Adalimumab ranked first, followed by bimekizumab, secukinumab q4w, and secukinumab q2w in terms of HiSCR, while bimekizumab showed superiority to adalimumab in DLQI 0/1 at weeks 12–16. Adverse effects were all similar to placebo.

Data regarding long-term efficacy and adverse effects are warranted. More RCTs for a single medication, greater numbers of participants, and consistent measurements are required to confirm the effects and safety of currently existing and new upcoming interventions.

## Figures and Tables

**Table 1:** Characteristics of included studies.

Source	Trial Name (Study Phase)	Inclusion Criteria	Follow-Up, Weeks	Treatment Arms: Patient Numbers	Age, Mean (SD)	Gender, Female (%)
Kimball et al., 2016 [21]	PIONEER I (Phase 3)	≥2 distinct anatomical areas, AN count of ≥3, IR to antibiotics	12	Adalimumab: 153 Placebo: 154	36.2 (10.8) 37.8 (11.3)	59.5 68.2
Kimball et al., 2016 [21]	PIONEER II (Phase 3)	≥2 distinct anatomical areas, AN count of ≥3, IR to antibiotics	12	Adalimumab: 163 Placebo: 163	34.9 (10.0) 36.1 (12.2)	66.3 69.3
Kimball et al., 2012 [22,23]	Nil (Phase 2)	Stable, moderate to severe HS	12	Adalimumab: 44 Placebo: 43	36.6 (10.7) 37.7 (12.0)	67.4 70.5
Bechara et al., 2021 [24]	SHARPS (Phase 4)	≥3 distinct anatomical areas, Draining fistula count of ≤20, AN count of ≥3	12	Adalimumab: 103 Placebo: 103	38.5 (11.7) 36.8 (10.8)	50 53
Kimball et al., 2022 [25]	SUNSHINE (Phase 3)	≥2 distinct anatomical areas, Draining fistula count of ≤20, AN count of ≥5	16	Secukinumab q4w: 180 Secukinumab q2w: 181 Placebo: 180	32.8 (7.9) 32.6 (7.9) 32.0 (7.1)	55.6 56.4 56.7
Kimball et al., 2022 [25]	SUNRISE (Phase 3)	≥2 distinct anatomical areas, Draining fistula count of ≤20, AN count of ≥5	16	Secukinumab q4w: 180 Secukinumab q2w: 180 Placebo: 183	32.0 (7.5) 31.9 (7.8) 31.4 (7.4)	57.2 54.4 57.4
Glatt et al., 2021 [26]	Nil (Phase 2)	Draining fistula count of ≤20, AN count of ≥3, IR to antibiotics	12	Bimekizumab: 44 Adalimumab: 20 Placebo: 20	36.7 (11.9) 31.1 (9.4) 40.7 (12.8)	65 81 67
NCT03926169 [27]	DETERMINED 1 (Phase 2)	≥2 distinct anatomical areas, Draining fistula count of ≤20, AN count of ≥5, IR to antibiotics	16	Risankizumab 180 mg: 80 Risankizumab 360 mg: 81 Placebo: 82	38.9 (11.5) 38.2 (12.0) 37.2 (12.0)	66.3 63.0 58.5
NCT03628924 [28]	NOVA (Phase 2)	≥2 distinct anatomical areas, Draining fistula count of ≤20, AN count of ≥3, IR to antibiotics	16	Guselkumab 200 mg sc: 59 Guselkumab 1200 mg IV, then 200 mg sc: 60 Placebo: 62	39.0 (12.4) 37.2 (10.9) 38.2 (11.6)	54.2 75.0 61.3
NCT03487276 [29]	SHINE (Phase 2)	≥ 2 distinct anatomical areas, AN count of ≥3, IR to antibiotics	16	IFX-1 800 mg Q4w: 35 Placebo: 36	35.0 (NA) 34.5 (NA)	51.4 58.3
Kirby et al., 2021 [30]	Aurora (Phase 2)	≥2 distinct anatomical areas, Draining fistula count of ≤20, AN count of ≥5, IR to antibiotics	12	Avacopan 30 mg bid: 134 Placebo: 130	NA	NA
Kimball et al., 2022 [31]	Nil (Phase 2)	≥2 distinct anatomical areas, Draining fistula count of ≤25, AN count of ≥4, BW 50 kg–150 kg	16	CJM 112: 33 Placebo: 33	36.0 (9.8) 39.0 (10.9)	66.7 66.7

**Table 2:** League table of HiSCR at 12–16 weeks.

**Adalimumab**	1.06 [0.68; 1.64]	.	.	.	.	.	.	.	.	.	1.79 [1.51; 2.12]
1.00 [0.66; 1.52]	**Bimekizumab**	.	.	.	.	.	.	.	.	.	2.27 [1.02; 5.07]
1.32 [1.03; 1.71]	1.32 [0.82; 2.13]	**Secukinumab** **q4w**	1.01 [0.85; 1.19]	.	.	.	.	.		.	1.35 [1.12; 1.63]
1.34 [1.04; 1.73]	1.34 [0.83; 2.15]	1.01 [0.86; 1.20]	**Secukinumab** **q2w**	.	.	.	.	.	.	.	1.34 [1.11; 1.62]
1.36 [0.88; 2.11]	1.36 [0.75; 2.47]	1.03 [0.66; 1.61]	1.02 [0.65; 1.59]	**Guselkumab 200 mg sc**	1.13 [0.78; 1.65]	.	.	.	.	.	1.31 [0.88; 1.96]
1.54 [0.98; 2.42]	1.54 [0.84; 2.82]	1.16 [0.73; 1.85]	1.15 [0.72; 1.83]	1.13 [0.78; 1.65]	**Guselkumab 1200 mg IV, then 200 mg sc**	.	.	.	.	.	1.16 [0.76; 1.77]
1.57 [1.07; 2.31]	1.57 [0.90; 2.74]	1.19 [0.80; 1.76]	1.17 [0.79; 1.74]	1.15 [0.68; 1.96]	1.02 [0.59; 1.76]	**Avacopan**	.	.	.	.	1.14 [0.81; 1.61]
1.61 [1.09; 2.37]	1.60 [0.92; 2.81]	1.21 [0.81; 1.81]	1.20 [0.80; 1.79]	1.18 [0.69; 2.01]	1.04 [0.60; 1.80]	1.02 [0.62; 1.67]	**Risankizumab 180 mg**	.	.	1.07 [0.76; 1.51]	1.12 [0.79; 1.58]
1.64 [0.98; 2.74]	1.63 [0.85; 3.14]	1.24 [0.73; 2.08]	1.22 [0.72; 2.06]	1.20 [0.64; 2.26]	1.06 [0.56; 2.02]	1.04 [0.57; 1.89]	1.02 [0.56; 1.86]	**IFX-1 800 mg Q4w**	.	.	1.09 [0.67; 1.78]
1.79 [0.93; 3.47]	1.79 [0.83; 3.88]	1.35 [0.69; 2.63]	1.34 [0.69; 2.60]	1.31 [0.62; 2.79]	1.16 [0.54; 2.50]	1.14 [0.55; 2.36]	1.12 [0.54; 2.31]	1.09 [0.49; 2.44]	**CJM112**	.	1.00 [0.53; 1.89]
1.72 [1.16; 2.56]	1.72 [0.97; 3.02]	1.30 [0.86; 1.95]	1.28 [0.85; 1.93]	1.26 [0.73; 2.16]	1.12 [0.64; 1.94]	1.09 [0.66; 1.80]	1.07 [0.76; 1.51]	1.05 [0.57; 1.92]	0.96 [0.46; 2.00]	**Risankizumab 360 mg**	1.04 [0.73; 1.49]
1.79 [1.51; 2.12]	1.79 [1.16; 2.77]	1.35 [1.12; 1.64]	1.34 [1.11; 1.62]	1.31 [0.88; 1.96]	1.16 [0.76; 1.77]	1.14 [0.81; 1.61]	1.12 [0.79; 1.58]	1.09 [0.67; 1.78]	1.00 [0.53; 1.89]	1.04 [0.73; 1.49]	**Placebo**

**Table 3:** League table of DLQI 0/1.

Pairwise Meta-Analysis			
**N** **M** **A**	**Bimekizumab**	2.25 [0.80; 6.40]	15.78 [0.99; 251.86]
	2.40 [0.88; 6.50]	**Adalimumab**	3.99 [1.71; 9.32]
	9.57 [2.75; 33.32]	3.99 [1.71; 9.32]	**Placebo**

**Table 4:** League table regarding the mean change of DLQI from baseline.

**CJM112**	.	.	.	−3.10 [−6.62; 0.42]
−0.40 [−4.56; 3.76]	**Guselkumab 200 mg sc**	−0.90 [−3.29; 1.49]	.	−2.70 [−4.92; −0.48]
−1.30 [−5.38; 2.78]	−0.90 [−3.29; 1.49]	**Guselkumab 1200 mg IV, then 200 mg sc**	.	−1.80 [−3.87; 0.27]
−2.00 [−6.85; 2.85]	−1.60 [−5.60; 2.40]	−0.70 [−4.62; 3.22]	**IFX−1 800 mg Q4w**	−1.10 [−4.43; 2.23]
−3.10 [−6.62; 0.42]	−2.70 [−4.92; −0.48]	−1.80 [−3.87; 0.27]	−1.10 [−4.43; 2.23]	**Placebo**

**Table 5:** League table presenting the NMA results of adverse effects.

**Risankizumab 360 mg**	.	.	1.17 [0.74; 1.84]	.	.	.	1.37 [0.84; 2.22]	.	.	.	.
1.03 [0.55; 1.93]	**Guselkumab 1200 mg IV, then 200 mg sc**	1.09 [0.76; 1.56]	.	.	.	.	1.32 [0.89; 1.96]	.	.	.	.
1.12 [0.60; 2.11]	1.09 [0.76; 1.56]	**Guselkumab 200 mg sc**	.	.	.	.	1.22 [0.81; 1.83]	.	.	.	.
1.17 [0.74; 1.84]	1.13 [0.59; 2.15]	1.04 [0.54; 1.99]	**Risankizumab 180 mg**	.	.	.	1.17 [0.71; 1.94]	.	.	.	.
1.32 [0.77; 2.25]	1.28 [0.81; 2.02]	1.18 [0.73; 1.88]	1.13 [0.65; 1.97]	**CJM112**	.	.	1.04 [0.82; 1.31]	.	.	.	.
1.33 [0.75; 2.34]	1.28 [0.78; 2.11]	1.18 [0.71; 1.97]	1.14 [0.63; 2.05]	1.01 [0.69; 1.47]	**Bimekizumab**	.	1.12 [0.76; 1.67]	0.97 [0.69; 1.37]	.	.	.
1.36 [0.83; 2.25]	1.32 [0.87; 2.00]	1.22 [0.79; 1.87]	1.17 [0.69; 1.97]	1.04 [0.79; 1.35]	1.03 [0.74; 1.43]	**Secukinumab q2w**	1.00 [0.88; 1.13]	.	1.01 [0.89; 1.15]	.	.
1.37 [0.84; 2.22]	1.32 [0.89; 1.96]	1.22 [0.81; 1.83]	1.17 [0.71; 1.94]	1.04 [0.82; 1.31]	1.03 [0.76; 1.39]	1.00 [0.88; 1.13]	**Placebo**	1.00 [0.90; 1.11]	1.01 [0.89; 1.14]	1.14 [0.89; 1.47]	1.20 [0.85; 1.71]
1.37 [0.83; 2.24]	1.32 [0.88; 1.99]	1.22 [0.80; 1.86]	1.17 [0.70; 1.97]	1.04 [0.80; 1.34]	1.03 [0.77; 1.39]	1.00 [0.85; 1.18]	1.00 [0.90; 1.11]	**Adalimumab**	.	.	.
1.38 [0.84; 2.27]	1.33 [0.88; 2.02]	1.23 [0.80; 1.89]	1.18 [0.70; 1.99]	1.05 [0.80; 1.36]	1.04 [0.75; 1.44]	1.01 [0.89; 1.15]	1.01 [0.89; 1.14]	1.01 [0.86; 1.19]	**Secukinumab q4w**	.	.
1.56 [0.91; 2.69]	1.51 [0.95; 2.41]	1.39 [0.86; 2.25]	1.34 [0.76; 2.35]	1.18 [0.84; 1.67]	1.18 [0.80; 1.74]	1.14 [0.87; 1.51]	1.14 [0.89; 1.47]	1.14 [0.87; 1.50]	1.13 [0.86; 1.50]	**Avacopan**	.
1.65 [0.91; 2.99]	1.59 [0.94; 2.70]	1.47 [0.86; 2.51]	1.41 [0.76; 2.61]	1.25 [0.82; 1.90]	1.24 [0.78; 1.97]	1.21 [0.83; 1.75]	1.20 [0.85; 1.71]	1.20 [0.83; 1.73]	1.19 [0.82; 1.73]	1.05 [0.69; 1.62]	**IFX-1 800 mg Q4w**

**Table 6:** Inconsistency evaluation of enrolled studies.

Comparison	Number of Studies	NMA	Direct	Indirect	Difference between Direct and Indirect Comparison	Lower Limit of 95% CI	Upper Limit of 95% CI	*p* Value
Bimekizumab: Adalimumab	1	−0.00118	−0.05449	0.408884	−0.46337	−1.76591	0.839167	0.485648
Bimekizumab: Placebo	1	0.581662	0.820981	0.480765	0.340215	−0.61613	1.29656	0.485648
Placebo: Secukinumab q4w	2	−0.30195	−0.29957	−1.60429	1.304712	−3.15112	5.76054	0.566037
Secukinumab q2w: Secukinumab q4w	2	−0.01153	−0.00972	−1.50507	1.495344	−3.30894	6.299623	0.541833

## Supplementary Materials

The following supporting information can be downloaded at: https://www.mdpi.com/article/10.3390/pharmaceutics15051351/s1, Table S1: Summary of the results of risk-of-bias; Table S2: Characteristics of excluded randomized controlled trials.
