# Supplementary material for: Efficacy and Safety of Biologics and Small Molecules for Moderate-to-Severe Hidradenitis Suppurativa: A Systematic Review and Network Meta-Analysis
Source: Pharmaceutics. 2023 Apr 28;15(5):1351. doi: 10.3390/pharmaceutics15051351 (PMC10224469; doi:10.3390/pharmaceutics15051351)
Supplement: Supplementary file 1 [file pharmaceutics-15-01351-s001.zip › pharmaceutics-2283677-supplementary.pdf]

Supplement Table S1: Summary of the results of risk-of-bias

Study	Medication	Outcome	Bias arising from the randomization process	Bias due to deviation from the intended intervention	Bias due to missing outcome data	Bias in the measurement of the outcome	Bias in selection of reported results	Overall risk of bias
Kimball et al. 2016 (PIONEER I)	Adalimumab	HiSCR, DLQI 0/1, AE	Low risk	Low risk	Low risk	Low risk	Low risk	Low risk
Kimball et al. 2016 (PIONEER II)	Adalimumab	HiSCR, DLQI 0/1, AE	Low risk	Low risk	Low risk	Low risk	Low risk	Low risk
Bechara et al. 2021 (SHARPS)	Adalimumab	HiSCR, AE	Low risk	Low risk	Low risk	Low risk	Low risk	Low risk
Kimball et al. 2012	Adalimumab	HiSCR, AE	Low risk	Low risk	Low risk	Low risk	Low risk	Low risk

Kimball et al. 2022 (SUNSHINE)	Secukinumab	HiSCR, AE	Low risk	Low risk	Low risk	Low risk	Low risk	Low risk
Kimball et al. 2022 (SUNRISE)	Secukinumab	HiSCR, AE	Low risk	Low risk	Low risk	Low risk	Low risk	Low risk
Glatt et al. 2021	Bimekizumab	HiSCR, DLQI 0/1, AE	Low risk	Some concern	Low risk	Low risk	Low risk	Some concern
NCT03926169 (DETERMINED 1)	Risankizumab	HiSCR, AE	Low risk	Low risk	Low risk	Low risk	Low risk	Low risk
NCT03628924 (NOVA)	Guselkumab	HiSCR, Mean DLQI, AE	Low risk	Low risk	Low risk	Low risk	Low risk	Low risk
NCT03487276 (SHINE)	IFX-1	HiSCR, Mean DLQI, AE	Low risk	Some concern	Low risk	Low risk	Low risk	Some concern

Kirby et al. 2021 (Aurora)	Avacopan	HiSCR, AE	Low risk	Low risk	Some concern	Low risk	Low risk	Some concern
Kimball et al. 2022	CJM 112	HiSCR, Mean DLQI, AE	Low risk	Some concern	Some concern	Low risk	Low risk	Some concern

Supplement Table S2. Characteristics of excluded randomized controlled trials

Target molecule	Source	Regimen	Follow-up, weeks	Treatment arms: patient numbers	Efficacy
TNFα	Grant et al. 2010 [28]	Infliximab 5 mg/kg iv at weeks 0, 2, 4, 6, 14, and 22	8	Infliximab: 15<br>Placebo: 23	27% versus 5%<br>achieving >50% decrease of HSSI<br>(p = 0.092)
TNFα	Adams et al. 2010 [29]	Etanercept 50 mg sc twice weekly	12	Etanercept: 10<br>Placebo: 10	PGA, pt-PGA, DLQI<br>(All p > 0.05)
IL-1	Tzanetakou et al. 2016 [30]	Anakinra 100 mg/day sc	12	Anakinra: 10<br>Placebo: 10	78% versus 30%<br>achieving HiSCR (p = 0.04)
IL-1	Kanni et al. 2018 [31]	Bermekimab (MABp1) 7.5 mg/kg iv every 2 weeks	12	Bermekimab: 10<br>Placebo: 10	60% versus 10%<br>achieving HiSCR (p = 0.035)
PDE-4	Vossen et al. 2019 [32]	Apremilast 30mg orally BID	16	Apremilast: 15<br>Placebo: 5	53.3% versus 0%<br>achieving HiSCR (p = 0.055)
JAK1	Alavi et al. 2022 [33]	INCB054707 90 mg orally QD	8	INCB054707: 8<br>Placebo: 9	88% versus 57%<br>achieving HiSCR

HSSI: HS Severity Index, PGA: physician global assessment, pt-PGA: patient global assessment
